# Entwicklungen in der Digitalisierung von Public Health seit 2020: Beispiele aus dem Leibniz-WissenschaftsCampus Digital Public Health Bremen

**DOI:** 10.1007/s00103-023-03827-9

**Published:** 2024-01-10

**Authors:** Hajo Zeeb, Benjamin Schüz, Tanja Schultz, Iris Pigeot

**Affiliations:** 1 Leibniz-Institut für Präventionsforschung und Epidemiologie-BIPS, Achterstr. 30, 28359 Bremen, Deutschland; 2 Leibniz-WissenschaftsCampus Digital Public Health Bremen, Bremen, Deutschland; 3 Institut für Public Health und Pflegewissenschaften, Universität Bremen, Bremen, Deutschland; 4 Cognitive Systems Lab, Universität Bremen, Bremen, Deutschland

**Keywords:** COVID-19, Digitale Biomarker, Digital Public Health, Fehlinformationen, Surveillance, COVID-19, Digital biomarker, Digital Public Health, Misinformation, Surveillance

## Abstract

Digital Public Health hat in den vergangenen Jahren insbesondere durch die mit der COVID-19-Pandemie verbundenen Anforderungen einen erheblichen Schub erfahren. In diesem Bericht geben wir einen Überblick über die Entwicklungen in der Digitalisierung im Bereich Public Health in Deutschland seit 2020 und illustrieren diese mit Beispielen aus dem Leibniz-WissenschaftsCampus Digital Public Health Bremen (LWC DiPH).

Zentral sind dabei folgende Themen: Wie prägen digitale Erhebungsmethoden sowie digitale Biomarker und Methoden der künstlichen Intelligenz die moderne epidemiologische und Präventionsforschung? Wie steht es um die Digitalisierung im öffentlichen Gesundheitsdienst? Welche Ansätze der gesundheitsökonomischen Evaluation von digitalen Public-Health-Interventionen wurden bisher eingesetzt? Wie steht es um die Aus- und Weiterbildung in diesem Bereich?

Auch die Arbeit des LWC DiPH war zunächst stark durch die COVID-19-Pandemie geprägt. Wiederholte populationsbezogene digitale Surveys des LWC DiPH ergaben Hinweise auf eine häufigere Nutzung von Gesundheitsapps in der Bevölkerung in Deutschland, z. B. bei den Anwendungen zur Unterstützung der körperlichen Aktivität. Dass die Digitalisierung von Public Health das Risiko von gezielten Fehl- und Desinformationen mit sich bringt, hat die COVID-19-Pandemie ebenfalls gezeigt.

## Abstract (en)

Digital public health has received a significant boost in recent years, especially due to the demands associated with the COVID-19 pandemic. In this report, we provide an overview of the developments in digitalization in the field of public health in Germany since 2020 and illustrate these with examples from the Leibniz ScienceCampus Digital Public Health Bremen (LSC DiPH).

The following topics are central: How do digital survey methods as well as digital biomarkers and artificial intelligence methods shape modern epidemiology and prevention research? What is the status of digitalization in public health offices? Which approaches to health economics evaluation of digital public health interventions have been utilized so far? What is the status of training and further education in digital public health?

The first years of the Leibniz ScienceCampus Digital Public Health Bremen (LSC DiPH) were also strongly influenced by the COVID-19 pandemic. Repeated population-based digital surveys of the LSC indicated an increase in use of health apps in the population, for example, in applications to support physical activity. The COVID-19-pandemic has also shown that the digitalization of public health enhances the risk of misinformation and disinformation.

## Hintergrund

Digital Public Health (DiPH) als der Versuch, digitale Entwicklungen und Technologien in Public-Health-Konzepte zu integrieren und so zur Erreichung von Public-Health-Zielen beizutragen, ist ein hochaktuelles und Zukunftsthema für die öffentliche Gesundheit und unser Gesundheitswesen. Das haben die Autorinnen und Autoren dieses Artikels in einem Überblicksartikel aus dem Jahr 2020 [1] beschrieben – und diese Einschätzung teilen wir auch heute noch.

Allerdings hat sich nicht zuletzt durch die COVID-19-Pandemie und dadurch angestoßene Entwicklungen eine neue Dynamik im Bereich digitaler Gesundheitstechnologien ergeben, was eine aktuelle Bestandsaufnahme und eine Systematik zur Beschreibung dieser Entwicklungen notwendig macht. Dieser Bericht fasst die Entwicklungen in diesem Bereich in Deutschland seit 2020 zusammen und illustriert sie am Beispiel von Arbeiten aus dem Leibniz-WissenschaftsCampus Digital Public Health Bremen (LWC DiPH).

Als eine Ordnungssystematik schlugen wir 2020 die Kategorisierung nach zentralen Public-Health-Funktionen entlang der Essential Public Health Operations der Weltgesundheitsorganisation (WHO) vor, zu denen auch die Überwachung von Krankheiten, die Beurteilung der Gesundheit der Bevölkerung und die Bestimmung vorrangiger Gesundheitsprobleme und Gesundheitsgefahren gehören [1]. Insbesondere die digitale Gestaltung dieser Public-Health-Funktionen erlangte in der sich ab März 2020 ausbreitenden COVID-19-Pandemie erhebliche Bedeutung. Die Pandemie hat insbesondere die Relevanz der Etablierung effektiver Monitoring- und Surveillancesysteme für verschiedene Public-Health-Ebenen hervorgehoben [2] und die Notwendigkeit einer umfassenden Digitalisierung im Bereich öffentlicher Gesundheit (und auch im Gesundheitswesen im engeren Sinne) demonstriert. Nicht zuletzt mit der weit verbreiteten und erfolgreichen, aber auch sozial unterschiedlichen Nutzung der Corona-Warn-App [3] und der Digitalisierung des COVID-19-Impfnachweises ab Anfang 2021 nahm auch die breite Bevölkerung die Vorteile und Chancen der digitalen Entwicklungen im Bereich Public Health wahr. Insbesondere befeuerte die ursprüngliche Entscheidung für einen zentralisierten Warn-App-Ansatz eine deutschlandweite Diskussion über Privatsphäre, Transparenz und Datensicherheit, drei Kernthemen der ethischen und rechtlichen Herausforderungen digitaler Werkzeuge in DiPH [*Schultz. T. et al. Chapter AI meets DiPH im Handbuch Digital Public Health, erscheint Anfang 2024*]. Der große Erfolgs- und Zeitdruck einer zuverlässigen Warn-App beschleunigte und fokussierte die Diskussionen und bewirkte schließlich ein Umdenken der Regierung zugunsten eines dezentralisierten Ansatzes. Der Warn-App-Code wurde allen Bürgerinnen und Bürgern öffentlich zugänglich gemacht. Die breite Akzeptanz der Warn-App und die hohe Zahl der freiwilligen Datenspenden zeigt, dass die Bevölkerung für die Einführung neuer digitaler Public-Health-Werkzeuge – zumindest in besonderen Situationen – offen ist. Zudem wurde die internetbasierte, zeitnahe Informationsvermittlung (insbesondere der grundlegenden epidemiologischen Datenlage z. B. durch die internationalen und nationalen Dashboards) zum ständigen Begleiter im Pandemiealltag. Gleichzeitig zeigte die Informationsvermittlung auch die Grenzen der bislang standardmäßig erhobenen und digital verfügbaren Daten auf. So verhinderte beispielsweise die Entscheidung, die COVID-19-Impfung nicht mit den Versichertendaten der gesetzlichen und privaten Krankenkassen zu verbinden, die Untersuchung der Impfeffekte in Langzeitstudien über Registerdaten. Mit der „Infodemie“, also dem Überfluss an Informationen einschließlich irreführender und falscher Informationen, traten zudem Risiken und unerwünschte Effekte der zunehmenden Digitalisierung auf, die die Komplexität der Entwicklung von Digital Public Health illustrierten.

Insgesamt kommen digitale Technologien und Anwendungen in den vergangenen Jahren im Bereich Public Health verstärkt zum Einsatz. Vor diesem Hintergrund diskutieren wir neuere Entwicklungen und Erkenntnisse zu Digital Public Health aus den 2020er-Jahren. Dabei steht vornehmlich Deutschland im Fokus. Mit LWC DiPH entstand Anfang 2020 eine interdisziplinäre Forschungsstruktur in Bremen, deren Entwicklung bis Ende 2023 zunächst orientierend nachgezeichnet wird. Im Anschluss zeigen wir den Einfluss der Digitalisierung auf verschiedene Bereiche der Public-Health-Forschung und in der Praxis auf. Dabei gehen wir insbesondere auf die Entwicklungen ein, die durch den Ausbruch der COVID-19-Pandemie vorangetrieben wurden. In einem Fazit arbeiten wir abschließend u. a. die Bedeutung von Fort- und Weiterbildung im Bereich Digital Public Health heraus.

## Entwicklung des LWC DiPH seit 2020

**Figure 1:**
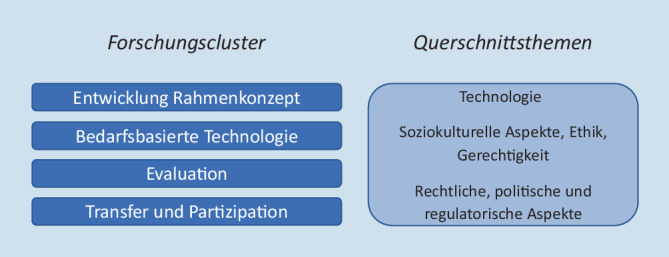

Der LWC DiPH wurde und wird (aktuell) im Rahmen einer kompetitiven Ausschreibung der Leibniz-Gemeinschaft gefördert und nahm zu Beginn der COVID-19-Pandemie seine Arbeit auf^1^. Entsprechend waren sowohl die Arbeitsweise als auch die initialen Inhalte vielfach von diesem akuten Public-Health-Problem geprägt. Mit seinen Forschungszielen rund um die theoretische Fundierung und nutzerbasierte Entwicklung digitaler Technologien für Public Health sowie die Evaluation und den partizipativen Forschungstransfer war der LWC DiPH thematisch an der Schnittstelle zentraler Herausforderungen der Pandemie positioniert. Die vier Forschungscluster des LWC DiPH sowie die querschnittlichen Themen (s. Abb. 1) bildeten die Grundlage dafür, COVID-19-bezogene Forschungsinhalte integrativ in das Arbeitsprogramm aufzunehmen. In enger Kooperation mit dem von vielen wissenschaftlichen Fachgesellschaften getragenen Kompetenznetz Public Health zu COVID-19 wurde schon im ersten Halbjahr 2020 ein umfassendes Hintergrundpapier zur Entwicklung von Contact Tracing Apps entwickelt [4]. Ein zentrales Ergebnis der interdisziplinären Arbeit im LWC DiPH war die iterative Entwicklung eines umfassenden Digital Public Health Frameworks (DigiPHrame, [5]). Neben empirischen Forschungsarbeiten zur digitalen Gesundheitskompetenz und der Nutzung digitaler Technologien für Prävention und Gesundheitsförderung liegt ein besonderer Fokus des LWC DiPH auf der Entwicklung und Evaluation von Digital-Public-Health-Interventionen, die auch das Thema der ersten internationalen Sommerschule im Juli 2023 waren. Zudem ist im Rahmen der ersten Förderphase des LWC DiPH (2019–2024) unter Beteiligung vieler LWC-Wissenschaftlerinnen und Wissenschaftlern in Zusammenarbeit mit nationalen und internationalen Autorinnen und Autoren ein englischsprachiges Handbuch Digital Public Health entstanden, das Anfang 2024 erscheint. Zu vielen der genannten Themen gibt es vertiefende Beiträge in der vorliegenden Ausgabe des Bundesgesundheitsblatts.

## Digitale Epidemiologie, künstliche Intelligenz und digitale Biomarker

Digitale Epidemiologie steht oftmals in enger Verbindung mit Neuerungen der Informationstechnologie, die bei der Verfügbarkeit und Erhebung von Daten relevant wird. Beispielsweise erzeugte die Notwendigkeit der Kontaktverfolgung zu Beginn der COVID-19-Pandemie eine Zusammenarbeit von großen IT-Providern wie Google und Apple, die das Google-Apple Exposure Notification (GAEN) Framework entwickelten, das erstmals Bluetooth-basierte Kontaktmessungen zwischen Smartphones verschiedener Betriebssysteme (iOS, Android) ermöglichte. Darüber hinaus erfordert die digitale Epidemiologie Neuentwicklungen von Methoden des maschinellen Lernens und der künstlichen Intelligenz, die für die Beschreibung, Analyse und Erklärung von dynamisch wachsenden, komplexen Datenmengen notwendig werden. Nachfolgend erläutern wir diese Dynamik anhand einiger Beispiele.

Die COVID-19-Pandemie bot für die digitale epidemiologische Forschung umfassende Herausforderungen und ein extrem vielseitiges Anwendungsfeld. Brockmann [6] sieht das Feld der digitalen Epidemiologie begründet in der detaillierten und immer umfassenderen Verfügbarkeit individueller Daten, insbesondere aufgrund der Verbreitung mobiler Endgeräte. Während der COVID-19-Pandemie konnten einerseits durch gezielte Datenspenden, andererseits über Bewegungsprofile sowie Analysen sozialer Medien wie Twitter und Facebook neuartige Erkenntnisse über Infektionsausbreitungen oder die Veränderung der Mobilität aufgrund von Public-Health-Maßnahmen zur Pandemiebekämpfung gewonnen werden [7].

Neben der offensichtlichen Anwendung von Methoden der künstlichen Intelligenz während der Pandemie gibt es weitere Anwendungsgebiete digitaler Epidemiologie. Dazu gehören z. B. die komplexe Abschätzung von COVID-19-Infektionsrisiken und die Erstellung prädiktiver Modelle mittels maschinellen Lernens aus Abwasseranalysen [8] oder technische Systeme zur physischen, kognitiven und sozialen Aktivierung von Menschen mit kognitiven Einschränkungen [9], die besonders zu Pandemiezeiten unter sozialer Deprivation und erhöhten Beziehungsbelastungen leiden [10]. Zudem werden neue Ansätze intensivierter longitudinaler Forschung zu psychischer Gesundheit erprobt [11, 12], die es beispielsweise erlauben, tagesgenau die Zusammenhänge zwischen Verhaltensempfehlungen und psychischer Gesundheit zu untersuchen. Diese neuen Forschungsansätze haben das Potenzial, die epidemiologische Forschung der Zukunft in erheblicher Weise zu verändern, indem neue Analyse- und Inferenzmöglichkeiten basierend auf der simultanen Anwendung maschineller Lernverfahren auf verschiedene Datenquellen zur Verfügung stehen werden.

In diesem Kontext werden zunehmend auch Sensordaten als „digitale Biomarker“ exploriert. Biomarker können sowohl vom menschlichen Körper erzeugte Biosignale wie etwa klinisch-physiologische Marker und neuronale Korrelate als auch geografische Parameter umfassen [13, 14]. Eine mit den Vorgaben der Europäischen Datenschutzgrundverordnung kompatible digitale Biomarker-Plattform (JTrack, [15]) bietet hier breite Möglichkeiten der Datensammlung und des integrierten Studienmanagements unter einem Open-Source-Rahmenkonzept. In der Sprachverarbeitung hat dieser Forschungsbereich bereits eine lange Tradition. Beispielsweise werden paralinguistische Merkmale aus Sprache automatisch extrahiert und in internationalen Challenges wie ComparE^2^ seit 2009 regelmäßig zur Einschätzung gesundheitsrelevanter Zustände (z. B. kognitive Einschränkungen, Atmung, Depressionen, Stress, COVID-19 etc.) evaluiert. Auch haben sich akustische und linguistische Merkmale aus longitudinalen Sprachaufzeichnungen von mehr als 1000 Teilnehmenden über 20 Jahre als sehr zuverlässige Marker zur Früherkennung kognitiver Einschränkungen erwiesen, die somit ggf. eine kostengünstige und niederschwellige Alternative zum flächendeckenden Screening bieten können [16].

Die personenbezogene primäre epidemiologische Forschung, etwa in der zunächst weitgehend analog aufgesetzten NAKO-Gesundheitsstudie, nutzt ebenfalls vermehrt digitale Technologien, sei es in der sensorbasierten Expositionsermittlung wie der Akzelerometrie, sei es in der über digitale Technologien gestützten Kohortenpflege oder auch nur in zusätzlichen digital durchgeführten Befragungen [17]. Einzelne größere epidemiologische Studien werden in Deutschland mittlerweile auch primär digital durchgeführt, mit der Möglichkeit, einwilligende Personen auch weitergehend zu persönlichen Terminen (z. B. zur Entnahme von Bioproben) einzuladen und dann weitergehend zu untersuchen. So wurde während der COVID-19-Pandemie die DigiHero-Studie der Universitätsmedizin und des Universitätsklinikums Halle aufgesetzt [18]. Bis Ende 2022 konnten schon über 80.000 Personen in die bundesweit rekrutierende Panelstudie eingeschlossen werden. Die digitale Studienbasis erlaubt auch zeitnahe Forschungen zu hochaktuellen gesundheitlichen Fragestellungen, wie etwa zu psychischen Auswirkungen des Krieges Russlands gegen die Ukraine [19]. Methodische Herausforderungen, etwa die sozial und altersbedingt ungleiche Teilnahme an derartigen Untersuchungen, werden für das Forschungsfeld der digitalen Epidemiologie weiterhin große Bedeutung haben. Diese Aspekte müssen einerseits genau untersucht werden [20], zum anderen gilt es, diesen Herausforderungen gezielt zu begegnen, etwa durch hybride oder nichtdigitale Teilnahmemöglichkeiten [21] und angepasste Stichprobenverfahren.

## Digital Public Health und der öffentliche Gesundheitsdienst

Zweifelsfrei hat sich auch die Arbeit der Gesundheitsämter während der COVID-19-Pandemie stark gewandelt. Mit der großen Zunahme an Bedeutung in der Infektionskontrolle, dem Informationsmanagement und vielen anderen Aufgaben rückte auch die oft noch wenig ausgeprägte Digitalisierung in das Blickfeld der Öffentlichkeit. Die engere digitale Zusammenarbeit der Gesundheitsämter mit dem Robert Koch-Institut über Softwareanwendungen wie das integrierte Fall- und Personenmanagementsystem SORMAS [22], neuere Versionen von Survnet und Survstat (zur Erfassung und Auswertung gemeldeter Infektionen) sowie das elektronische Meldesystem DEMIS [22] weisen aber darauf hin, dass dieser Bereich sehr dynamisch vorangetrieben wurde und die Digitalisierung der Public-Health-Aufgaben von Gesundheitsämtern mittlerweile eine Routineentwicklung darstellt. Forschungen des LWC DiPH trugen während der COVID-19-Pandemie zu diesen Entwicklungen bei: In einer Zusammenarbeit des Fraunhofer Zentrums für Techno- und Wirtschaftsmathematik mit Partnern des LWC DiPH (Fraunhofer MEVIS und Leibniz-Institut BIPS) wurde z. B. zu einer Softwareanwendung zur Entscheidungsunterstützung in Gesundheitsämtern geforscht und diese gemeinsam mit Praktikerinnen und Praktikern aus Gesundheitsämtern entwickelt und erprobt [23]. Diese Anwendung basiert auf fünf verschiedenen statistischen Modellen, die literaturbasiert entwickelt wurden und für spezifische Fragestellungen zu individuellen oder Gruppen-Infektionsverläufen Aussagen generieren. Durchgeführt wurde die methodische Entwicklungsarbeit parallel zu Nutzerevaluationen unter anderem mittels Fokusgruppeninterviews und konkreten, auf den kognitiven Aufwand bei der Nutzung fokussierten Anwendungsanalysen (sog. Cognitive Walkthroughs).

Im Pakt für den öffentlichen Gesundheitsdienst (ÖGD) ist die Digitalisierung einer der Schwerpunkte, der bis 2026 von der Bundesregierung mit ca. 800 Mio. € unterstützt werden soll. Der erste Bericht des Beirats des Pakts ÖGD [24] nennt als erforderliche Maßnahmen der ÖGD-Digitalisierung neben einer Bedarfserhebung innerhalb des ÖGD den weiteren Ausbau von DEMIS sowie eine Vereinheitlichung und verbesserte Interoperabilität der Systeme für den Infektionsschutz und andere Aufgabenbereiche. Auch die Anbindung der Gesundheitsämter an die Telematikstruktur des Gesundheitswesens wird gefordert. Vielfältige Projekte sollen zur Verwirklichung des digitalen Gesundheitsamts als Zielvorstellung beitragen. Erste Ergebnisse einer Erhebung und Evaluation der digitalen Reife von Gesundheitsämtern wurden im Juli 2023 veröffentlicht. Dabei zeigte sich, dass gemäß der Selbsteinschätzung der Gesundheitsämter in acht beurteilten Dimensionen im Median überwiegend nur die unteren beiden Entwicklungsstufen des fünfstufigen Reifegradschemas erreicht wurden [25]. Allerdings gibt es auch einige Gesundheitsämter, die schon sehr stark digitalisiert sind und somit Beispielfunktion übernehmen können. Insgesamt sind im ÖGD in den vergangenen Jahren umfangreiche Prozesse angestoßen worden, die in der Zukunft eine deutlich verbesserte Grundlage für die weitere Entwicklung von Digital Public Health im Kontext des ÖGD bieten.

## Digital Public Health in der Bevölkerung: Surveyergebnisse im Zeitverlauf

Zum Monitoring der Entwicklung von Digital-Public-Health-Aspekten in der Bevölkerung wurden durch den LWC DiPH zwei panelbasierte computerassistierte telefonische Surveys in den Jahren 2020 und 2022 durchgeführt, die sich an internetnutzende Personen richteten. Im ersten Survey [26] wurde neben Fragen zur COVID-19-Pandemie und allgemein zur Digitalisierung das Thema körperliche Aktivität durch fünf Fragen bearbeitet. Zudem wurde digitale Gesundheitskompetenz mit dem eHEALS-Tool (dt. Version, [27]) gemessen. 57 % der 991 Teilnehmenden (mittleres Alter 55 ± 16 Jahre) gaben an, digitale Technologien für gesundheitsbezogene Zwecke zu nutzen. Ganz überwiegend herrschte die Erwartung vor, dass Digitalisierung bedeutsam sowohl für Therapie und Gesundheitsversorgung (89 %) als auch für Gesundheitsförderung (69 %) sein wird. Etwas mehr als ein Drittel der grundsätzlich Digitaltechnologie-Nutzenden gaben an, Gesundheitsapps einzusetzen oder dies zu planen und gut 30 % davon nutzten ihren Angaben zu Folge digitale Technologien zur Förderung der körperlichen Aktivität.

**Figure 2:**
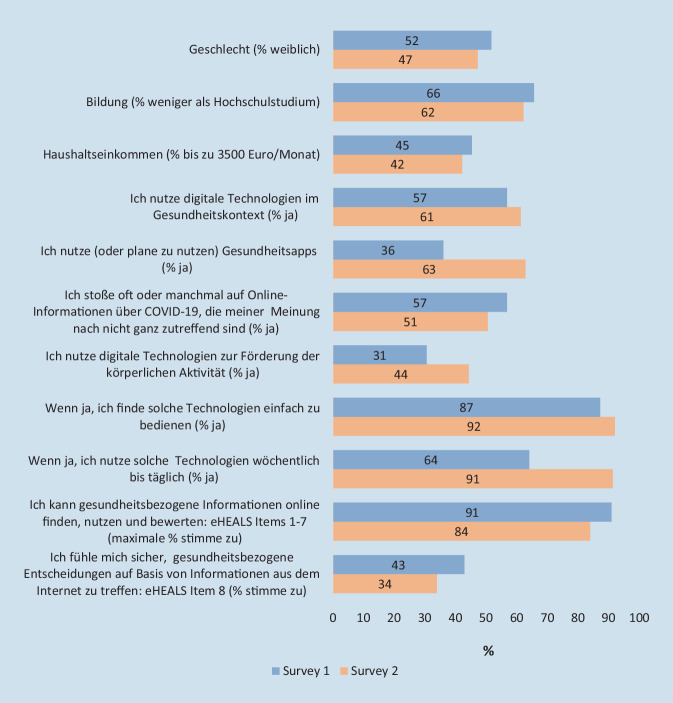

Zwei Jahre später befragten wir erneut 1020 Erwachsene (mittleres Alter 56 ± 16 Jahre) mit vergleichbarer Methodik [*De Santis et al., submitted*]. Eine Nutzung digitaler Technologien im Gesundheitskontext gaben nun 61 % der Befragten an. Der Anteil derjenigen, die diese Nutzung im Kontext der Förderung körperlicher Aktivität nannten, lag nun bei über 44 % und damit fast 14 Prozentpunkte über dem Wert von 2020. Auch die Gesundheitsapp-Nutzung (bzw. eine geplante Nutzung) hat deutlich zugelegt: Sie stieg auf nunmehr knapp 63 % unter denjenigen, die überhaupt Digitaltechnologie für die Gesundheit einsetzen. Etwas überraschend sind allerdings die niedrigeren Werte des zweiten Surveys in Bezug auf einzelne Komponenten des eHEALS-Fragebogens zur digitalen Gesundheitskompetenz, die im Widerspruch zu anderen Untersuchungen stehen. Abb. 2 zeigt den Vergleich der beiden Surveys zu ausgewählten Aspekten und Fragestellungen.

Für beide Surveys gilt, dass aufgrund des Zugangs über ein internetbasiertes Panel keine Personen ohne Internetzugang und damit außerhalb der Digitalität eingeschlossen wurden. Allerdings betrifft dies in Deutschland nur noch sehr wenige Menschen (5 % der Personen im Alter von 16 bis 74 Jahren), darunter anteilig mehr Ältere [28]. Im zeitlichen Vergleich geben die Surveys recht deutliche Hinweise darauf, dass die Nutzung digitaler Technologien für die Gesundheit innerhalb weniger Jahre weiter zugenommen hat und insbesondere Gesundheitsapps mittlerweile deutlich an Popularität gewonnen haben. Dabei sind Apps zur körperlichen Aktivität eine wichtige Anwendung; allerdings war während der COVID-19-Pandemie eine besonders große Nutzung der Corona-Warn-App und weiterer COVID-19-Apps zur Information oder für andere Zwecke zu verzeichnen. Digitale Technologien werden aber auch in erheblichem Maße für die Information zu chronischen Erkrankungen und ihren Ursachen eingesetzt, wie gut 20 % der Befragten im Survey des Jahres 2022 in Bezug auf Krebs angaben.

## Informationen und Falschinformationen: Digitale Kommunikation über Gesundheit

Zu den essenziellen Funktionen von Public Health gehört auch die Vermittlung von gesundheitsrelevanten Informationen. In diesem Zusammenhang hat die gesundheitsbezogene Analyse sozialer Netzwerkdaten als Teilbereich der digitalen Epidemiologie während der COVID-19-Pandemie besondere Aufmerksamkeit erfahren. Die während der Pandemie von der WHO und anderen internationalen Institutionen als Infodemie [29] beschriebene Informationsflut mit einer extremen Dynamik der Informations- und Desinformationsangebote insbesondere in den sozialen Medien wurde zu einem eigenen Forschungsgegenstand, auch wenn der Begriff „Infodemiology“ schon im Jahr 2002 geprägt wurde [30]. Angesichts weiterer Public-Health-Krisen und zukünftiger Pandemien ist von einer wachsenden Bedeutung entsprechender Forschungen auszugehen.

Die Potenziale vor allem digitaler Medien zur Informationsverbreitung auch in schwer erreichbare Zielgruppen wurden bereits vor der Pandemie betont [31]. Auch die Risiken der Verbreitung von gesundheitsbezogenen Falschinformationen wie MMR-Impfmythen [32] oder zu den Ursachen von AIDS [33] wurden bereits vor COVID-19 diskutiert. Allerdings hat sich durch die Ubiquität der Pandemie und der Schutzmaßnahmen während der Pandemie das Ausmaß an gesundheitsbezogenen Falschinformationen in den sozialen Medien deutlich erhöht [34]. Zentrale Herausforderungen liegen darin, auf der einen Seite evidenzbasierte Informationen rechtzeitig und zielgenau an die Adressatengruppen zu bringen, für die sie relevant und teilweise auch zeitkritisch sind, zum anderen aber auch, Fehlinformationen zu entkräften und idealerweise deren Verbreitung vorzubeugen.

Um der Verbreitung von Fehlinformationen in sozialen Netzwerken vorzubeugen, wurden zum einen Maßnahmen entwickelt, die Fehlinformationen inhaltlich entkräften sowie Nutzende von sozialen Netzwerken auf die Exposition von Fehlinformationen vorbereiten und zum kritischen Lesen von Informationen anregen. So zeigen beispielsweise Studien, dass Informationen darüber, wie Falschinformationen aufgebaut und zu erkennen sind, die Anfälligkeit gegenüber Fehlinformationen und deren Verbreitung verringern können [35]. Zum anderen kann die Identifikation von Determinanten des Teilens von Fehlinformationen dazu beitragen, Aspekte der Infodemie zu bearbeiten, die nicht auf den Inhalten von Informationen, sondern auf kontextuellen Bedingungen beruhen, beispielsweise soziale Aspekte oder Eigenschaften der jeweiligen Person. Im Rahmen des LWC DiPH wurden dazu Scoping-Reviews durchgeführt [36, 37], die zeigen, dass neben den Inhalten auch bestimmte Eigenschaften wie etwa Gesundheitskompetenz oder das Bedürfnis nach kognitiver Beanspruchung erklären können, ob Menschen Fehlinformationen glauben und weiterverbreiten. In einer Interventionsstudie konnte zudem gezeigt werden, dass zusätzlich zu den bekannten Maßnahmen zur Hervorhebung von Fehlinformationen soziale Mechanismen wie normative Informationen dazu beitragen können, die Verbreitung von Fehlinformationen zu reduzieren [38].

## Digitalisierung in der kommunalen Prävention und Gesundheitsförderung

Auch in diesem Bereich war die Entwicklung digitaler Technologien in den letzten Jahren entscheidend durch die COVID-19-Pandemie geprägt. Maßgeblich hierfür waren die wiederholten Kontaktbeschränkungen und damit die Notwendigkeit für Anbieter kommunaler Prävention und Gesundheitsförderung, auf digitale Technologien zu setzen, um vorhandene Angebote in gewissem Rahmen aufrechtzuerhalten oder neue digitale Maßnahmen einzubringen. Im Breitensport entwickelte die Mehrzahl der Vereine digitale Angebote; sie mussten dabei aber sowohl mit technischen als auch mit inhaltlichen Schwierigkeiten umgehen, wie etwa der fehlenden Möglichkeit zur direkten Unterstützung im persönlichen Kontakt [39]. Auch die Landesvereinigungen für Gesundheit und die Volkshochschulen mit ihren vielfältigen gesundheitsbezogenen Angeboten stellten sich in ihrer Arbeitsweise und dem Angebotsspektrum auf digitale Projekte und Kommunikation um. Allerdings konnten trotz des Digitalisierungsschubs viele Public-Health-relevante Leistungen z. B. in der Beratung, Vorsorge und Früherkennung, bei denen ein direkter persönlicher Kontakt notwendig ist, nicht digital aufgefangen werden oder sie nahmen an Qualität ab [39]. In den kommenden Jahren wird sich zeigen, inwieweit der Einsatz digitaler Technologien in diesem Bereich beibehalten oder sogar weiterentwickelt wird, wenn die besonderen Rahmenbedingungen durch COVID-19 nicht mehr bestehen. Eine digitale Kompetenzentwicklung bei vielen Akteurinnen und Akteuren und Institutionen hat zweifelsfrei stattgefunden und auch die digitale Gesundheitskompetenz der Allgemeinbevölkerung hat sich zumindest in einigen Gruppen verbessert [40].

## Digitale Public-Health-Interventionen: ökonomische Evaluation

Nur für wenige digitale Public-Health-Interventionen liegen bisher ökonomische Evaluationen vor und diese beziehen sich zumeist auf digitale Maßnahmen zu verhaltensbezogenen Risikofaktoren und deren Prävention. Eine im Rahmen des LWC DiPH durchgeführte Übersichtsarbeit schloss 14 Studien aus den Jahren 2009–2022 ein, die sich mit evaluierten digitalen Interventionen zum Zweck der Primärprävention oder Gesundheitsförderung beschäftigten [41]. Bei insgesamt sehr limitierter Evidenzbasis aufgrund unzureichender Studiendaten war eine der Schlussfolgerungen, dass es Hinweise auf kosteneffektive digitale Public-Health-Interventionen gibt, allerdings noch umfangreiche methodische Herausforderungen bei der Bewertung der Effektivität und damit auch bei der Kosten-Nutzen-Bewertung von digitalen Public-Health-Interventionen bestehen. Insgesamt bleibt die Zahl entsprechend evaluierter Evaluationen auch heute noch sehr klein, sodass wissenschaftlich überzeugende Aussagen zu den ökonomischen Aspekten von digitalen Public-Health-Interventionen auch im Jahr 2024 kaum möglich sind.

## Aus- und Weiterbildung in digitaler Public Health

Auch im Bereich Aus- und Weiterbildung besteht ein starkes interdisziplinäres Interesse an Anwendungen von Digital Public Health, deren Effekten und Konsequenzen. Dies zeigt sich beispielsweise in der zunehmenden Zahl von Veranstaltungen zu dieser Thematik im Rahmen der jährlichen europäischen Public-Health-Konferenzen. Im deutschen Kontext fanden 2023 mehrere internationale Sommerschulen statt (z. B. der Sommerworkshop an der Universität Bayreuth zum Thema digitale Spaltung und Ungleichheiten in Gesundheitsförderung und Public Health). An der internationalen Sommerschule des LWC DiPH im Juli 2023 beschäftigten sich über 30 Teilnehmende aus 13 Nationen und mit unterschiedlichem wissenschaftlichem Hintergrund (aus einem Pool von knapp 80 Bewerbungen) intensiv mit wissenschaftlichen und technischen Grundlagen und Konsequenzen digitaler Interventionen und entwickelten eigene Konzepte im Rahmen fiktiver, gleichwohl realistischer Anwendungsszenarien. Die höchst positive Bewertung der Sommerschule durch die Teilnehmenden legt nahe, dass weitere derartige Angebote folgen sollten und ein starkes interdisziplinäres Interesse an Anwendungen von Digital Public Health, deren Effekten und Konsequenzen besteht. Gleichzeitig zeigt dieses hohe Interesse, dass es einen Bedarf an angepassten oder neu entwickelten Studieninhalten und Studiengängen in diesem Bereich gibt. Aktuell sind Inhalte, Kompetenzen und Fertigkeiten im Bereich digitaler Public Health noch nicht flächendeckend in die Curricula der gesundheitswissenschaftlichen Studiengänge an den deutschen Hochschulen integriert (siehe auch Beitrag von Albrecht und Maaß et al. in diesem Themenheft). Für die kommenden Jahre besteht erheblicher Handlungsbedarf, um in diesem wichtigen Zukunftsbereich auch international anschlussfähig zu bleiben.

## Fazit

Mit der COVID-19-Pandemie war für den Bereich Public Health seit 2020 ein starker Digitalisierungsschub verbunden, der unter anderem dazu führte, dass gesundheitsbezogene Apps wie die Corona-Warn-App oder digitale Impfnachweise weite Verbreitung und Akzeptanz in der Bevölkerung fanden. Digitale Kommunikationsmedien wurden zunehmend wichtig für die Informationsvermittlung und den Austausch sowie für viele Aspekte des Infektionsschutzes. Allerdings wurden sie auch für Desinformationskampagnen genutzt. Gleichzeitig zeigte sich an vielen Stellen, wo die Umsetzung weitreichender Digitalisierung im Gesundheitswesen notwendig ist und wo gleichzeitig die größten Hürden für Innovation und Umsetzung liegen. Die digitale Epidemiologie als Grundlage von (Digital) Public Health lieferte nicht nur zahlreiche Erkenntnisse zur Pandemie, sondern auch neuartige Visualisierungen grundlegender epidemiologischer Daten, die für die moderne Public-Health-Kommunikation wichtige Impulse geben. Digital Public Health ist ein dynamisches und gerade in den vergangenen Jahren stark expandierendes Feld, das auch neue Anforderungen in Bezug auf interdisziplinäres Arbeiten und moderne Ausbildungskonzepte mit sich bringt.

## Footnotes

1 Leibniz-Einrichtungen und Hochschulen können sich um Leibniz-WissenschaftsCampi (sic) bewerben, um in regionalen Partnerschaften zusammenzuarbeiten und den jeweiligen Forschungsbereich weiterzuentwickeln. Siehe www.leibniz-gemeinschaft.de/forschung/leibniz-wissenschaftscampi.

2 https://signalprocessingsociety.org/publications-resources/data-challenges/computational-paralinguistics-challenge-compare.
